# The Feasibility of Using the Direct Anterior Approach for Total Hip Arthroplasty or Bipolar Hemiarthroplasty to Treat Femoral Neck Fractures among the Elderly

**DOI:** 10.1155/2022/2115586

**Published:** 2022-07-20

**Authors:** Shigeo Ishiguro, Kunihiro Asanuma, Tomohito Hagi, Hidehiko Ohsumi, Hiroki Wakabayashi, Akihiro Sudo

**Affiliations:** ^1^Kameyama City Medical Center, Orthopaedic Surgery, Mie, Japan; ^2^Department of Orthopaedic Surgery, Mie University Graduate School of Medicine, Mie, Japan; ^3^Ohsumi Orthopedic Surgery & Surgery of the Hand, Japan

## Abstract

**Purpose:**

Femoral neck fractures (FNFs) are a significant cause of mortality and disability among the elderly. Total hip arthroplasty (THA) is the preferred treatment method in active, cognitively intact patients. In less active or cognitively impaired patients, bipolar hemiarthroplasty (BHA) is the practical option in Japan. Even with the direct anterior approach (DAA), clinical concerns about conducting THA in elderly patients include possible dislocations, critical complications, and medical cost-effectiveness. This study is aimed at rethinking the practical surgical indications for FNFs.

**Methods:**

Between April 2019 and March 2021, BHA patients with displaced FNF (*n* = 21) performed through the DAA were compared with THA patients with displaced FNF (*n* = 19). The perioperative complications, clinical and radiologic outcomes, and mortality were compared between groups retrospectively at six months.

**Results:**

THA patients had an increased average operation time (103.3 min vs. 89.1 min, *P* < 0.05) and similar amounts of bleeding (183.16 ml. vs. 121.1 ml.). The percentages of patients who received biological transfusion showed no difference, with low rates of perioperative complications (4% vs. 0%) and similar mortality rates compared to BHA patients. One THA patient experienced posterior dislocation during a state of postoperative delirium.

**Conclusion:**

THA through the DAA might be a credible and safe option for FNF patients, with excellent functional outcomes and fewer surgery-related complications. Early posterior dislocation might be related to optimized offset distance and not related to leg discrepancy or other radiographic items. Hence, orthopedic surgeons should reconsider their options before conducting BPH for elderly and cognitively intact FNF patients.

## 1. Introduction

Femoral neck fracture (FNF) is a significant cause of morbidity and disability among the elderly [1, 2]. Early fracture treatment improves survival, reduces hospital stay, and restores long-term functional capacity [3]. Not only do patients undergoing surgery for FNF have a higher risk of mortality and postoperative complications, but surgical choices and indications on femoral neck fractures also vary depending on the country [4–6]. Despite growing support for the use of THA in the management of acute FNFs, concerns regarding potential increased blood loss, operation duration, and dislocation risks remain. Some authors recommend the use of technology such as dual mobility components to reduce the risk of dislocation after THA [7], the use of a specific leg positioner, or the use of an image intensifier [2]. As FNFs will continue to increase, ordinary hospitals will have to manage them. In ordinary institutions, however, cutting-edge devices or intraoperative radiographic evaluation are not always affordable or practical.

Although recent studies suggest excellent outcomes with the DAA in FNF patients undergoing hemiarthroplasty [8, 9], there are little available data in the literature regarding the outcomes of patients treated with THA through the DAA [2]. Therefore, the purpose of this study is (1) to compare clinical outcomes, perioperative complications, and short-term mortality through the DAA between THA and BHA in FNF patients (Garden classification 3 or 4) and (2) to identify the patient- and surgery-associated factors that could potentially influence surgical outcomes. The implants used for the two series in this study were a cementless acetabular component (Delta-TT, Japan *Lima* Co), or bipolar head and cementless femoral stem (Master SL, Japan *Lima* Co), installed with either standard or lateralized offset according to the preoperative plan.

## 2. Methods

This study was approved by both our institution's internal review board and its ethics committee. Each patient was properly informed of the known differences between THA and BHA and provided written consent. If a patient was cognitively impaired, the decision for consent was left to the unanimous decision of family members. This study was conducted entirely at the first author`s institution. In our institution, all THAs and BHAs were performed through the DAA. All patients who presented to our emergency department with displaced (Garden 3 or 4) FNF and no additional injuries, and who received THA or BHA, were enrolled in this study.

### 2.1. Patient Characteristics

We retrospectively reviewed the medical records of all THA and BHA patients with a displaced (Garden 3-4) FNF from April 2019 to March 2021. We then compared them and conducted a subanalysis. Perioperative data included operative time and estimated blood loss. Baseline characteristics including age, gender, BMI, and the patients` physical status according to ASA were also recorded (Table 1).

### 2.2. Surgical Technique and Postoperative Care

All cases (both THA and BHA) were performed via a minimally invasive DAA in a supine position using the ordinary surgical table (Belmont, Hyogo Prefecture, Japan), under general anesthesia, and with a femoral nerve block of 20 cc of 0.75% ropivacaine. In THA cases, the anterior aspect of the joint capsule and the whole acetabular labrum were resected completely to expose the bony structure of the acetabulum. The cementless acetabular component was always fixed with either 2 or 3 screws. In BHA cases, the joint capsule was incised in an inverse T shape and the capsule around the intertrochanter area was partially resected. In both of the series, the posterior aspect of the joint capsule was partially detached from its insertion to the posterior aspect of the greater trochanter when the proximal part of the femur was elevated for the femoral stem insertion. In the trial reduction, first, it was confirmed that full flexion in neutral rotation did not provoke posterior dislocation. Next, it was confirmed that 90 degrees of hip flexion with forced internal rotation until resistance was felt manually did not provoke posterior dislocation. Intraoperative imaging was not used at any point in the procedure. In both series, when closing the fascia of the tensor fascia lata, 20 ml of 10% transamin was scattered under the fascia. Implants used in this study for these two series were either a cementless acetabular component (Delta-TT, Japan *Lima* Co) or bipolar head and cementless femoral stem (Master SL, Japan *Lima* Co), with the use of standard or lateralized offset determined by the preoperative plan. However, if intraoperative joint laxity or dislocatability was found, the preoperative plan was amended.

Beginning on the first postoperative day, all patients followed a standardized physical therapy protocol with mobilization out of bed, progressing from walker to no assistive devices as tolerated.

For prevention of deep vein thrombosis, an elastic bandage was kept on for 10 days postoperatively and no additional medication was prescribed.

### 2.3. Clinical Evaluation

The patients received clinical and radiographical follow-ups at regular intervals according to their needs. We could not evaluate clinical function using either the Harris Hip Score (HHS) or JOA Score (Japanese Orthopaedic Association Score) due to the inclusion of patients with diminished physical states and/or other degenerative orthopedic disorders in the populations of these series. As a result, we clarified the ambulatory states into four categories (Table 2).

### 2.4. Radiologic Evaluation

For the preoperative and postoperative radiographs, the standardized protocol was applied.

The patient was placed in a supine position and the lower limbs were held together in a neutral position. On the anterior-posterior view of the pelvis, the leg length discrepancy (LLD) was measured as the perpendicular distance between a line passing through both teardrop points medial to the acetabula and the corresponding tip of the lesser trochanter [10].

### 2.5. Statistical Analysis

All the statistical analyses were performed using SPSS version 27 software (SPSS Inc., Chicago, IL).

## 3. Results

### 3.1. Patient Characteristics

A total of 40 FNF patients (male with BHA: 5, female with BHA: 16, male with THA: 4, female with THA: 15) with an average age of 83 years (range 70–96) were identified (Table 1). The average duration between hospital admission and surgery was 7 days. The average follow-up period was 11 months (range 6–27). Besides the comparison between THA and BHA, subanalyses between gender and with or without cognitive impairment were conducted.

### 3.2. Intraoperative Complication and Perioperative Parameters

For THA patients, the average operation time was 14 minutes longer (*P* < 0.05) than the BHA group (Table 1), with intraoperative blood loss averaging 62 ml higher for the THA group (not significant). No intraoperative fractures occurred among the 40 patients in this study.

### 3.3. Hospital Stay and Mortality

**Figure 1:**
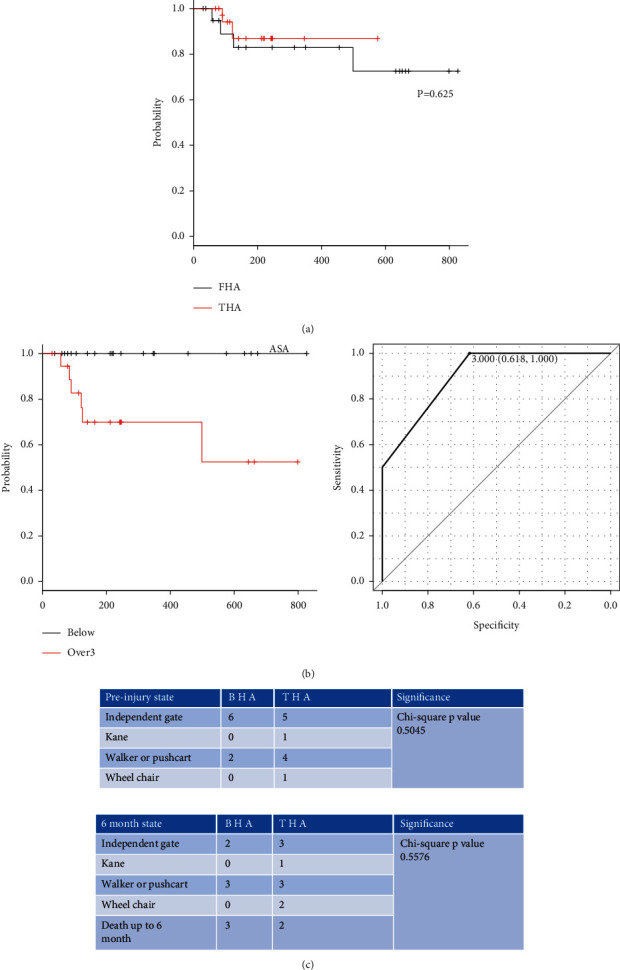
(a) Kaplan–Meier survival analysis showed no statistical difference between the THA group and the BHA group. (b) To confirm the diagnostic accuracy, ROC analysis was performed by evaluating the area under the curve (AUC). The AUC for identifying death was 0.904 (95% CI 0.811–0.998). Based on the ROC analysis, a cut-off value was the ASA score. The score of 3 and above was defined as the risk-high group, and the score of 2 and below was defined as the low-risk group to clarify the survival rate. (c) In performing chi-square analysis, there was no increased death in the THA group even in ASA 3 and above.

The average hospital stay durations for the BHA and THA groups were 57 days and 51 days, respectively (not significant). The duration of hospital stay tended to correlate with patient age (*r* = 0.294, *P*=0.0693); however, it did not show any correlation to ASA score, or days to operation. The 30-day mortality rate of both groups was 0%. At six-month follow-up, three patients out of 21 in the BHA group had died and two out of 19 in the THA group had died (Table 3) (Figure 1).

### 3.4. Complication Rate and Revision

One THA patient experienced posterior dislocation while in a postoperative delirious state; however, it was managed with closed reduction and did not require revision surgery. This patient had slight cognitive impairment, with normal offset, and an optimal prosthesis setting.

At an average follow-up of 11 months, there were no deep infections, no wound dehiscence, no other dislocations, no periprosthetic fracture, and no reoperations (Figures 2 and 3).

### 3.5. Functional Outcomes, Cognition Impairment, and Radiographic Findings

The average deterioration of the functional score was less among THA patients with normal cognition than in cognitively impaired THA patients (*P*=0.0141). Among cognitively normal patients, functional aggravation among THA patients tended to be less than that of BHA patients (*P*=0.10). Between BHA patients with or without cognition impairment, the functional results were comparable.

Comparing normal offset and lateralized offset, or between an offset ratio of greater or less than 1, neither functional difference nor the range of motion differences were observed (Table 4).

## 4. Discussion

THA is the preferred treatment for displaced FNF in active, cognitively intact patients. However, THA is accompanied by an increased risk of joint dislocation [11] and higher medical costs. In this aging society, the number of patients with osteoporosis and cognitive impairment is increasing and it must be premediated.

Previous papers reported that THA in FNF patients might be a technically more challenging procedure, as it is associated with increased intraoperative blood loss, longer operation times, and higher short-term mortality. The mortality rate in this pilot study was, however, lower than that reported in the literature [2,12]. No significant increase was seen in the surgery-related complication rate between THA patients and their BHA counterparts except for acute posterior dislocation [2]. Unlike the previous literature, increased BMI and ASA scores were not correlated with duration of hospital stay and admission medical cost.

In this study, one acute posterior dislocation occurred. Plausible reasons for this are temporary postoperative delirium in addition to cognitive impairment and/or lack of adequate tension of the reconstructed joint. Some authors preserve the anterior aspect of the joint capsule and not excising it, even when THA is performed through the DAA [2]. By doing so, the necessary tension to prevent dislocation must be preserved. In skeletally small patients like those in this series, however, operative maneuvers for acetabular bone are extremely difficult to perform, especially in cases where bone quality is poor and subclinical joint contracture exists. Hence, the surgical procedure is accompanied by an increased risk of intraoperative fracture if the anterior aspect of joint capsule is preserved.

In the THA group, as femoral offsets are over 6 mm in 4 cases, this seems excessively large compared to the ideal offset for arthroplasty [10,13]. Little et al. mentioned in their 5-year follow-up that the polyethylene wear rate was 0.12 mm/year in the group whose offset was within 5 mm of normal and 0.16 mm/year in the counterpart group whose offset was over 5 mm of normal. Their study group, however, was in their sixties at the time of operation. Hence, it would follow that their activity levels differed from that of our patients.

During the THA surgeries in this study, maximum care was taken to avoid postoperative dislocation. If operation team members had concerns about this, the simplest way to increase tension was to use a lateralized offset prosthesis, under the condition that no leg length discrepancy existed. This explains why femoral offset in the THA group is always greater than that of the nonfracture side. In 4 cases, the offset distance was over optimal as we were not allowed to use a navigation or tension measuring system. Taking into consideration the life expectancy of the population of this study group, a minor increase in the polyethylene wear rate might be permissible.

In terms of medical costs, hospital stay, complication rate, and life expectancy after surgery, no notable differences existed between the THA and BHA groups. Hence, cognitively intact patients who sustain a displaced FNF should receive THA if the situation permits, after proper informed consent.

In the early 2020s, cement usage in trochanteric fractures has been growing in popularity, or in cases with rare pathologies [14]. In this series, all patients were treated without cement usage; however, we admit that some cases might present better clinical outcomes with cement usage [15]. To preserve the safety of patients, a slow and prudent introduction of new ideas is always necessary.

## 5. Conclusion

THA through the DAA might be a credible and safe option for FNF patients. This method produces excellent functional outcomes and fewer surgery-related complications. The early posterior dislocation might be related to optimized offset distance and not related to leg discrepancy or other radiographic items. This study has a limitation due to the small sample size. We believe, however, that the sample demographics of this study closely match the demographics of patients suffering from FNFs in Japan as a whole and therefore may be representative of the larger population. Further study is therefore warranted. Orthopedic surgeons should reconsider before deciding to conduct BPH for elderly and cognitively intact FNS patients [16].

## Figures and Tables

**Table 1:** Patient characteristics.

Parameters	BHA (*n* = 21)	THA (*n* = 19)	Significance
BMI (kg/m^2^)	30.4 (6.6)	31.3 (6.6)	NS
Age (y)	84 (6.0)	81 (7.2)	NS
Gender (*n*)			
Male	5	4	NS
Female	16	15	NS
ASA score (*n*)			
1	2	0	*P*=0.0421
2	11	8	
3	5	11	
4	3	0	
Aggravation of functional score (given in Table 2)	0.611	0.471	NS
Intraoperative blood loss	121 (142)	183 (148)	NS
Operation duration	89 (19.3)	103 (13.9)	*P* < 0.05

**Table 2:** Ambulatory state score.

Independent gait	1
Kane	2
Walker or pushcart	3
Wheel chair	4

**Table 3:** Fatal complications in BHA group and THA group within 6 months postoperatively.

	Cause of death		Survival period
BHA	Urethral cancer	Already diagnosed at surgery	2 months
BHA	Hemodialysis	Already diagnosed at surgery	5 months
THA	Undifferentiated lung cancer	Diagnosed at death	4 months
THA	Acute pancreatitis	Diagnosed before death	3 months
BHA	Senility	Diagnosed before death	3 months

**Table 4:** Radiographic findings.

Parameters	BHA (*n* = 21)	THA (*n* = 19)	Significance
Offset of fracture side	62.9 (4.9)	69.7 (4.5)	*P* < 0.0001
Offset of nonfracture side	64.6 (6.4)	66.1(4.7)	NS
Offset difference	−1.7 (6.3)	3.6 (4.8)	*P* < 0.01
Leg length discrepancy	4.7 (5.9)	3.7 (3.8)	NS

## Data Availability

The datasets used and/or analyzed in the current study are available from the corresponding author on reasonable request.

## Additional Points

Orthopedic surgeons can reconsider before deciding to conduct BHA for elderly FNS patients who is cognitively intact and belongs to ASA 1 or 2.
